# What is the appropriate surgical strategy for pulmonary metastasis of colorectal cancer?

**DOI:** 10.1097/MD.0000000000021368

**Published:** 2020-07-24

**Authors:** Hang Li, Hong Hu, Bin Li, Xiangjie Sun, Yihua Sun, Haiquan Chen

**Affiliations:** a Department of Thoracic Surgery and State Key Laboratory of Genetic Engineering, Fudan University Shanghai Cancer Center; b Institute of Thoracic Oncology; c Department of Oncology, Shanghai Medical College, Fudan University; d Department of Pathology, Fudan University Shanghai Cancer Center, Shanghai, China.

**Keywords:** colorectal cancer, pulmonary metastasectomy, surgery extend, systematic mediastinal lymph nodes dissection

## Abstract

Pulmonary metastasectomy is considered to be a feasible method for selected colorectal cancer (CRC) patients. This study aimed to optimize the individualized surgical strategy of pulmonary metastasectomy, especially in choice of surgery extent and systematic mediastinal lymph nodes dissection.

Data of 267 CRC patients who underwent pulmonary metastasectomy from July 2011 to July 2017 in Shanghai Cancer Center Fudan University were reviewed. Recurrence-free survival (RFS), overall survival (OS) and other clinical characteristics were compared between patients who accepted different surgical strategy.

A total of 93 (34.8%) patients underwent lobectomy, 162 (60.7%) wedge resection, and 12 (4.5%) segmentectomy. Mediastinal lymph nodes dissection or sampling was performed in 106 (39.7%) patients. The median follow-up phase was 32.5 months (range 7.2–104.7 months). Patients were divided into 2 groups according to the surgical extent, lobectomy group and sublobar resection group. The median RFS and OS were 46.4 and 76.5 months for patients underwent, respectively. In the patients whose tumor diameter was ≥ 1.5 cm, RFS (5-year; 44.9% vs 29.8%, log-rank *P* = .03; hazard ratio, 0.71; 95% CI 0.52–0.89, *P* = .026) was better in the lobectomy group; however, no difference was found in OS. Meanwhile, in the patients whose tumor size was <1.5 cm, no difference was observed in RFS, as well as in OS. In the patients with metastatic lesion size ≥1.5 cm, a trend towards better RFS was found in patients received lymph nodes dissection, but it did not reach statistical significance.

Lobectomy has more curative significance for CRC patients with single pulmonary metastatic lesion ≥1.5 cm. Systematic mediastinal lymph nodes dissection did not improve clinical outcome for CRC patients occurred pulmonary metastasis.

## 1 Introduction

Pulmonary metastasectomy (PM) is considered to be a feasible method for selected colorectal cancer (CRC) patients with a limited number of metastases and sufficient pulmonary function. The 5-year overall survival rates of patients who underwent surgical resection have been reported to range from 27% to 68%.^[1–4]^ A randomized controlled trial concerning the value and extent of PM in patients with CRC metastases is currently performed;^[5]^ meanwhile some retrospective studies had concluded that sufficient resection margin, such as lobectomy, could provide benefits for survival.^[6,7]^ However, some pulmonary metastases are sub-centimeter lesions, for which lobectomy might be too much.

The incidence of mediastinal lymph node metastases in pulmonary metastases is reported to range from 12% to 19%, positive mediastinal lymph node metastases have been demonstrated to be associated with poorer survival.^[8–13]^ Although systematic dissection or sampling of mediastinal lymph nodes could provide further understanding of metastatic disease staging, its value in improving survival still need to be investigated.

Therefore, this study aimed to optimize the individualized surgical strategy for CRC patients having pulmonary metastases, especially concerning choice of surgery extent of resection and systematic mediastinal lymph nodes dissection.

## 2 Patients and methods

### 2.1 Patients

The data of patients were respectively collected from the patients’ chart of Shanghai Cancer Center Fudan University from July 2011 to July 2017. Inclusion criteria for this study were:

1) patients received pulmonary surgery with radical intention, which meant, no apparent macroscopic lesion was left;
2) patients had history of resected CRC and the excised pulmonary lesion should be considered metastasis of the earlier CRC;
3) pulmonary metastasis was single lesion;
4) re-review pathological diagnosis of metastatic CRC was confirmed by 2 pathologists according to the results of morphology and immunohistochemical staining. Written informed consent was received from all patients. This study was approved by the ethics committee in our institute.

### 2.2 Clinical features

Clinical characteristics including gender, age at diagnosis, tumor size, surgical technique and extent, presence of lymph nodes dissection and metastases, tumor location were collected from the archives in our institute. Patients were inquired every 3 months after the date of surgery either in clinic or by telephone about disease recurrence and survival information. Recurrence-free survival (RFS) was calculated from date of PM to date of recurrence or last-time follow-up. Overall survival (OS) was defined as the time elapsed between date of PM to date of death or last-time follow-up.

### 2.3 Statistical analyses

We applied Pearson *Χ*^*2*^ test or Fisher exact tests to do bivariate analysis of clinical characteristics. Survival curves were made using Kaplan–Meier method. Relapse-free survival and overall survival were compared using log-rank test. Cox Regression was performed to assess the correlation between clinicopathological characteristics and survival. The statistical analyses were performed using SPSS 16.0 for Windows (Chicago, IL). All tests were 2 tailed, and statistical significance was set as *P* < .05.

## 3 Results

### 3.1 Patient characteristics

From January 2008 to September 2016, 11,022 patients received surgery in department of thoracic surgery. According to the inclusion criteria, 267 patients were collected for the study. After PM, all the patients received chemotherapy recommended by the multidisciplinary team. Among these patients, 108(40.4%) were female and 159 (59.6%) were male, and ranged in age at diagnosis from 30 to 81 (median 59) years old. The median tumor size was 2.0 cm, ranging from 0.7 cm to 9 cm. 44.6% (119 out of 267) patients had tumor in the left lung and 55.4% (148 out of 267) in the right side. Lobectomy was performed in 93 (34.8%) patients, wedge resection in 162 (60.7%), and segmentectomy in 12 (4.5%) patients. Mediastinal lymph nodes dissection or sampling was performed in 106 (39.7%) patients. Lymph node metastases occurred in 13 patients, accounting for 12.3% of the patients who underwent lymph nodes dissection or sampling. The number of patients receiving surgery by video-assisted thoracoscopic surgery or muscle-sparing open technique was 160 (59.9%) and 107 (40.1%), respectively (Table 1 and Table 2).

**Table 1:**
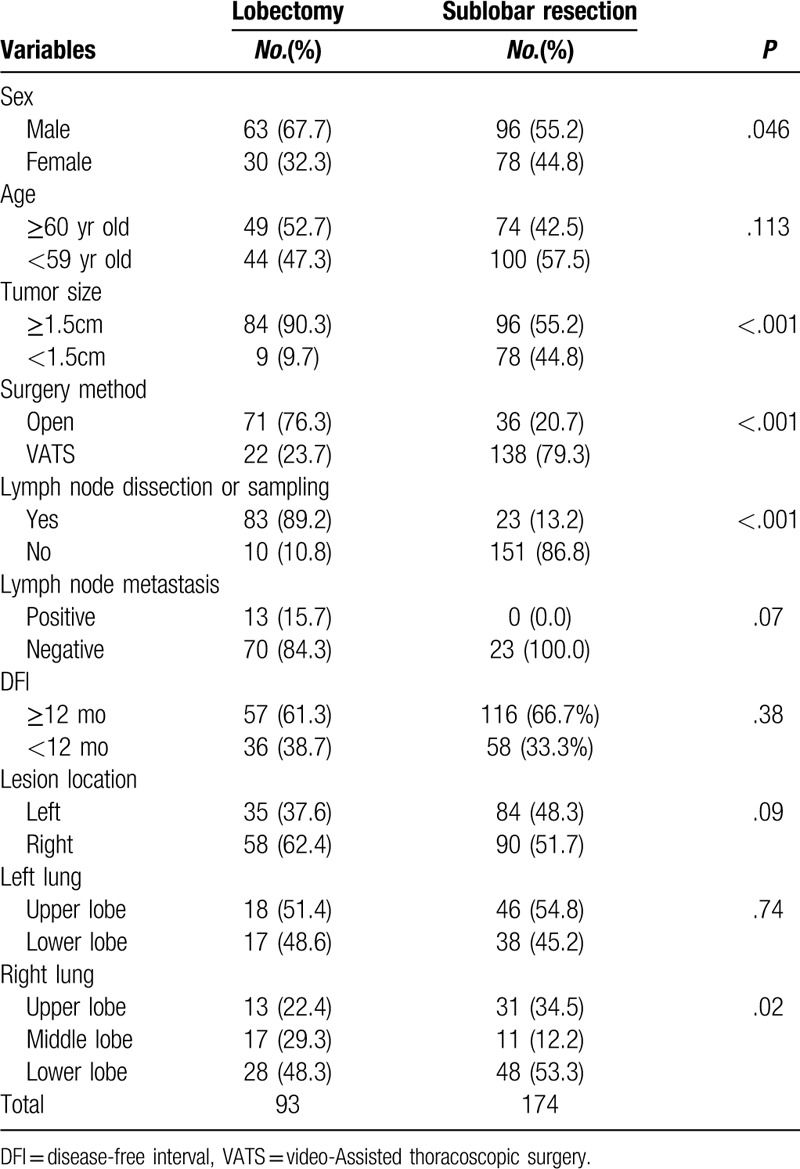
Clinical features of patients underwent lobectomy or sublobar resection.

**Table 2:**
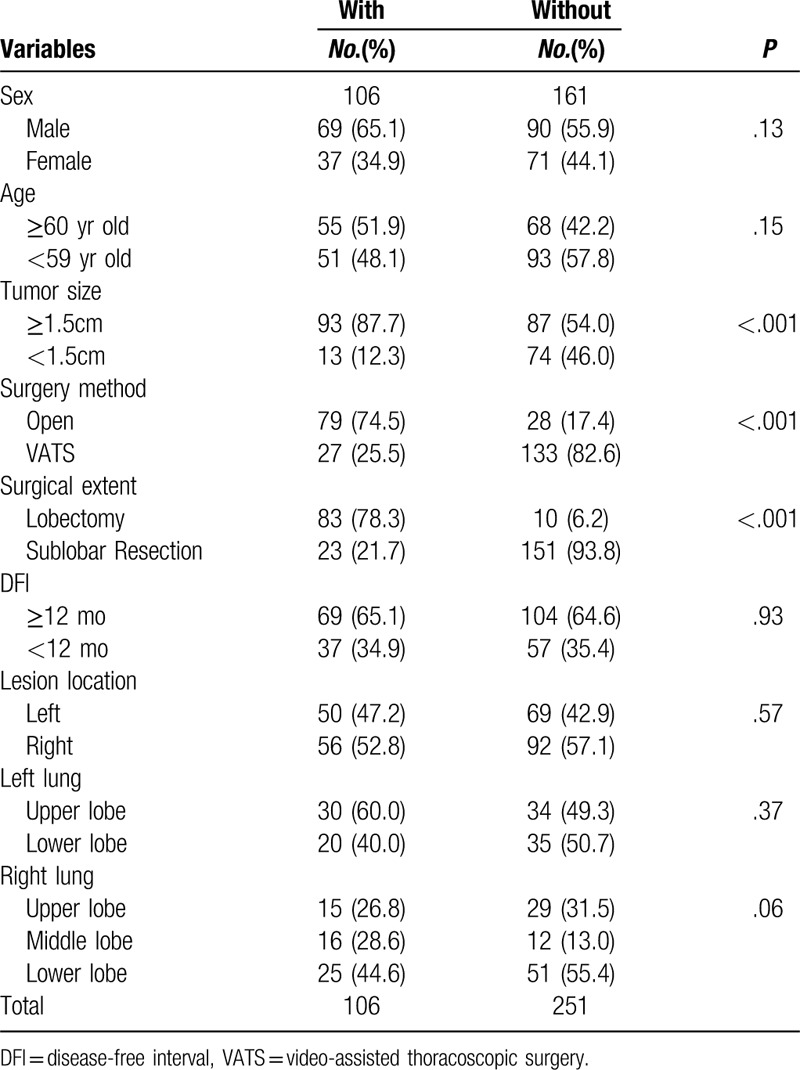
Clinical features of patients with or without systematic mediastinal lymph nodes dissection or sampling.

Bivariate analysis of clinical characteristics was performed in all patients. No remarkable differences in gender, age, disease-free interval and tumor location were found between patients with lobectomy and those with sub-lobar resection. Significantly more patients received lymph nodes dissection (89.2% vs 13.2%, *P* < .001) and open surgery (76.3% vs 20.7%) in lobectomy group. In the patients that received lymph nodes dissection or sampling, no remarkable differences were found in age, gender, and disease-free interval, neither.

### 3.2 Surgical extent and prognosis

**Figure 1:**
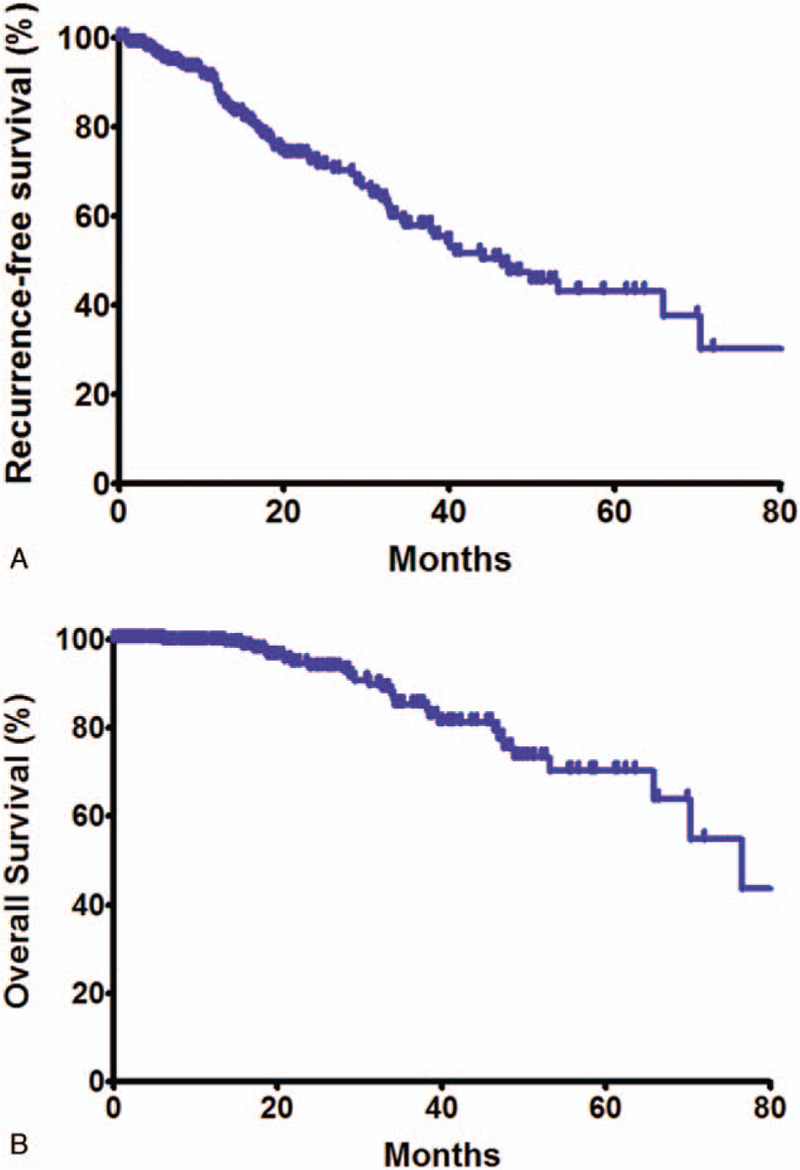
Recurrence-free survival and overall survival of colorectal cancer patients underwent pulmonary metastasectomy.

**Figure 2:**
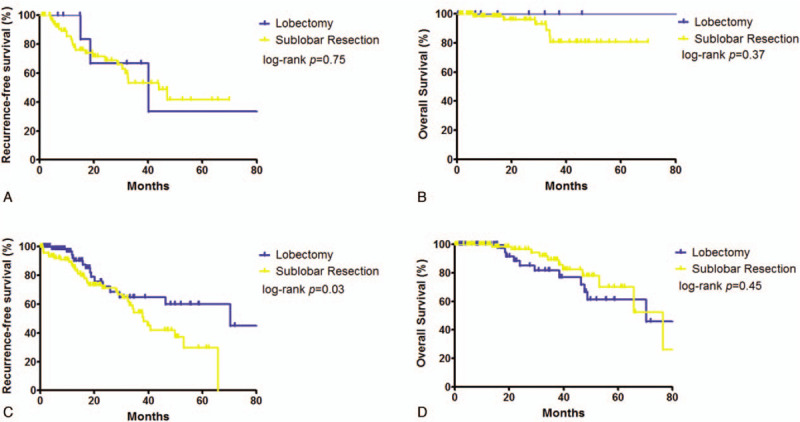
Recurrence-free survival and overall survival according to the extent of resection (lobectomy vesus sublobar resection); A and B, in patients with tumor less than 1.5 cm; C and D, in patients with tumor over 1.5 cm (1.5 cm included).

The median follow-up period was 32.5 months (range 7.2–104.7 months). The median RFS and OS was 46.4 and 76.5 months, respectively (Fig. 1). Clinical outcome of patients who received lobectomy vs those who received sub-lobar resection was further investigated. In the subgroup analysis, patients were divided by the tumor size. In the patients whose tumor diameter was ≥ 1.5 cm, RFS (5-year; 44.9% vs 29.8%, log-rank *P* = .03; hazard ratio, 0.71; 95% confidence interval, 0.52–0.89, *P* = .026) was better in the lobectomy group; however, no difference was found in OS (5-year; 61.2% vs 70.0%, log-rank *P* = .45). Meanwhile, in the patients whose tumor size was < 1.5 cm, no difference was observed in RFS (5-year; 33.3% vs 41.2%, log-rank *P* = .75), nor in OS (5-year; 100% vs 80.6%, log-rank *P* = .37, Fig. 2).

**Figure 3:**
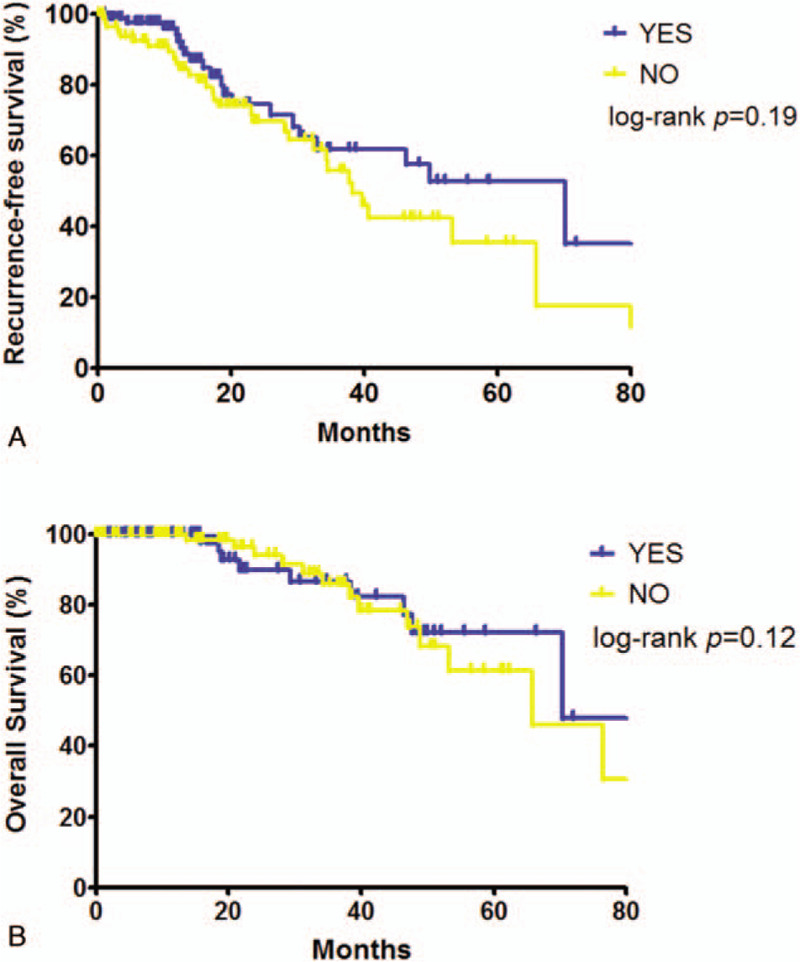
Recurrence-free survival and overall survival according to the type of lymphnode dissection (with versus without systematic mediastinal lymphadenectomy) in patients with tumor size over 1.5 cm (1.5 cm included).

Prognosis of patients with or without lymph nodes dissection were investigated. There was no difference in the survival between patients with and without systematic mediastinal lymphadenectomy. In the patients with metastatic lesion size ≥ 1.5 cm, a trend towards better RFS was found in patients who underwent lymph nodes dissection, but this did not reach statistical significance (5-year RFS; 52.7% vs 35.4%, log-rank *P* = .19, Fig. 3).

## 4 Discussion

In the last decades, pulmonary metastasectomy has been accepted as a therapeutic option for colorectal cancer patients according to the guidelines of the National Comprehensive Cancer Network.^[14]^ However, appropriate surgical extent and the indication for mediastinal lymph nodes dissection are still controversial issues.

In the present study, lobectomy led to more favorable RFS when compared with sub-lobar resection in patients with a single metastatic lesion ≥ 1.5 cm. Several previous studies have suggested that major resection, such as lobectomy, might benefit CRC patients who undergo pulmonary metastasectomy.^[6,15]^ However, from a theoretical point of view major resection will compromise pulmonary function. For some patients esp. having a sub-centimeter metastatic lesion, a sublobar resection might suffice. We carried out this investigation with an attempt to find out which subgroup of patient would benefit from lobectomy.

With regard to mediastinal lymph nodes dissection, our data showed that mediastinal lymph nodes dissection or sampling did not improve survival in CRC patients who underwent pulmonary metastasectomy. Some previous studies showed the outcome of CRC patients was negatively associated with mediastinal lymph nodes metastases.^[8–10]^ However, systematic lymph nodes dissection did not result in a remarkable survival benefit in the patients with positive mediastinal lymph nodes; neither a better overall survival, nor a prolonged nodal recurrence-free period was achieved.^[16]^ It seems that aggressive lymphadenectomy does not result in better survival, and it did not change the following multidisciplinary therapy.

There are several limitations of our study. It is a non-randomized retrospective study and has the selection bias of patients. The follow-up phase is not long enough. We investigated only OS because our data did not allow us to analyze cancer-specific survival. More detailed and precise results may be revealed in the future as the continuous collection of patients and clinical data. Furthermore, a randomized trial should be designed to validate the findings of this study.

In conclusion, we found that lobectomy probably has more curative significance than sublobar resection for CRC patients with pulmonary metastatic lesion over 1.5 cm. Systematic mediastinal lymph nodes dissection did not improve clinical outcome for CRC patients with a pulmonary metastasis. Thus, sub-classification and individualized surgical treatment could have clinical benefit for CRC patients with a single pulmonary metastasis.

## Author contributions

HL and HH were in charge of the data collection and analysis. HL and BL were in charge of the manuscript writing. XS was in charge of pathological examination. YS and HC were in charge of surgical procedure. HC was in charge of the study design.

**Methodology:** Hong Hu.
